# It Is All about Pressure

**DOI:** 10.3390/jcm11133640

**Published:** 2022-06-23

**Authors:** Paolo Brusini, Maria Letizia Salvetat, Marco Zeppieri

**Affiliations:** 1 Department of Ophthalmology, Policlinico “Città di Udine”, 33100 Udine, Italy; brusini@libero.it; 2 Department of Ophthalmology, Azienda Sanitaria Friuli Occidentale, 33170 Pordenone, Italy; mlsalvetat@hotmail.it; 3 Department of Ophthalmology, University Hospital of Udine, 33100 Udine, Italy

Glaucoma is an ocular disease caused by elevated intraocular pressure that leads to progressive optic neuropathy. The irreversible morphologic and functional damage is characterized by progressive visual field loss and retinal ganglion cell degeneration. Glaucoma tends to be a silent disease, in which central visual acuity is only affected at late stages; if left untreated, it can lead to blindness. The prevalence of this disease worldwide is more than 70 million, which is thought to increase to over 100 million by the year 2040 [1,2]. The main risk factor for primary open-angle glaucoma (POAG) and other forms of glaucoma, which is also the target for therapy, is elevated intraocular pressure [3,4]. Local medical therapy, laser, and surgical treatments are all geared to lowering IOP to reduce the incidence and progression of glaucoma [5,6]. Thus, it is of the utmost importance that all patients, especially those with risk factors, undergo periodic ophthalmic examinations that include tonometry. The primary goal of this Special Issue entitled “Intraocular Pressure and Ocular Hypertension” is to provide a collection of pertinent topics and highlight the importance of IOP, tonometry, aqueous humor (AH) dynamics, trabecular meshwork (TM) outflow pathways, and treatment options in glaucoma and ocular hypertension (OHT).

Aqueous production and outflow are both involved in IOP regulation; however, most glaucomatous conditions, such as POAG, are characterized by reduced outflow. The TM plays an important role in the disease process. IOP homeostasis is influenced by aqueous humor outflow, which is characterized by a pulsatile flow pattern evident in Schlemm’s canal and in the TM pathway [7]. Du et al. [8] reported that phase-sensitive optical coherence tomography demonstrates that pulsatile movement of the TM tends to be reduced in POAG patients, especially those with greater diurnal IOP fluctuations. TM has shown to be more rigid and less flexible in glaucomatous patients. This type of innovative diagnostic testing method has the potential of being of clinical use when deciding on treatment options in those glaucoma patients that do not reach IOP target levels and show progression of disease, which may require more aggressive treatment and/or prompt surgery.

Given that the IOP measurement is a fundamental part of any complete ophthalmological examination, numerous instruments, known as tonometers, have been proposed in order to obtain IOP measurements [9,10]. The measurement of the true IOP value in vivo requires invasive intraocular manometry. All tonometric methods available on the market just provide an estimation of IOP. The evaluation of the precision and accuracy of the different tonometers and the identification of the variables that can influence a correct IOP measurement represent an important field of research.

Our Review article, entitled “How to measure intraocular pressure: an update review of various tonometers” [11], describes the different instruments used to measure the IOP through the ages. Even if the Goldmann applanation tonometer (GAT) is still considered the gold standard technique in measuring IOP, several other instruments based on different operating principles (indentation, applanation, rebound, contour matching) have been proposed. The manuscript highlights advantages and drawbacks of the various devices, emphasizing the concept that the continuous monitoring of IOP, which is still under evaluation, will be an important step in the diagnosis and management of the glaucomatous patients.

In the article entitled “Intraocular pressure measurement in childhood glaucoma under standardized general anesthesia: the prospective EyeBIS study”, Alicja Strzalkowska et al. [12] addresses the important topic of the IOP measurement in children. The authors compared the IOP measurements taken with the iCare PRO rebound tonometer and Perkins applanation tonometer in glaucomatous and healthy children (mean age of 45 ± 30 months) under general anesthesia. The results of the study demonstrated that the IOP values taken with both tonometers appeared inversely related to the anesthesia depth, and that iCare IOP values were significantly higher than those obtained with Perkins tonometer in both glaucomatous and healthy children.

Several studies have compared the performances of different tonometers, and how different variables can affect the accuracy. Sugihara and Tanito [13] analyze the effects of aging on IOP measured by three different tonometers. Corneal biomechanical properties change with age, which can differently influence the IOP measurement obtained with different devices. Comparing the IOP measurements taken with GAT, non-contact tonometer, and rebound tonometer, the authors found that age appeared negatively correlated with the IOP values measured with non-contact and rebound tonometers, whereas GAT IOP measurements were not influenced by age.

The Ocular Response Analyzer (ORA) was used by Jóźnik et al. [14] to assess the biomechanical behavior of the cornea after a water drinking test in patients with or without a previous XEN Gel implant. They found a significant difference between the two groups in various analyzed parameters, results indicating that ORA could be useful in postoperative glaucoma diagnostics. Moreover, Diaz-Barreda et al. [15] reported significant modifications in corneal biomechanical parameters measured with ORA in patients that underwent a deep sclerectomy with Esnoper V2000 implant. Corneal hysteresis remained above preoperative values at 3 months of follow-up, whereas corneal resistance factor was at a lower level. The clinical relevance of this information, however, needs to be confirmed with further studies.

Another paper regarding the correlation between glaucoma surgery and corneal properties was written by Onoe et al. [16]. These authors found a significant increase in corneal higher-order aberrations after ab interno trabeculotomy or goniotomy performed with the Kahook Dual Blade combined with phacoemulsification. These findings should be considered when planning this type of surgical procedures. Moreover, patients should be informed about this possible complication prior to surgery.

In a further study, Okada et al. [17] did not find any significant difference in the outcomes in patients operated with phacoemulsification associated either with a 120° or 180° incision of the Schlemm’s canal performed by means of an ab-interno trabeculotomy. Given that the same group in the study cited above found an increase of corneal aberrations following an extensive incision of Schlemm’s canal, the authors suggest to preferably perform a 120° incision during an ab-interno trabeculotomy.

The effects of treatment on anatomic structures can be helpful in better understanding physiologic pathways and in the discovery of new mechanisms to treat diseases. The paper entitled “The dual effect of Rho-kinase inhibition on trabecular meshwork cells cytoskeleton and extracellular matrix in an in vitro model of glaucoma” [18] shows that Rho-kinase inhibitor can have an effect on the cytoskeleton organization and extracellular matrix of the TM, thus providing new insights to TM outflow pathway mechanisms involved in glaucoma that can be of clinical interest in the development of treatments for elevated IOP.

The causes of glaucoma are multifactorial and in part still unknown. Several risk factors like family history, thin cornea, African American race, ocular hypertension, etc. are known; however, factors associated with the manifestation of the disease have yet to be discovered. Maddala et al. addressed this lacuna by looking at the levels of growth/differential factor-15 (GDF15) in the AH and serum samples in patients with glaucoma and age- and gender- matched controls [19]. The study showed significant and important serum and AH levels of GDF15 in patients with POAG when compared to controls. The paper entitled “Serum Calcium Level as a Useful Surrogate for Risk of Elevated Intraocular Pressure” [20] also looked at possible factors associated with glaucoma, and found that high serum total calcium levels were significantly associated with elevated IOP in a large cohort of Asians. Studies like the ones reported here can help find potential biomarkers for the diagnosis, management and prognosis of glaucoma, in addition to providing better understanding of the physiological pathway mechanisms involved in the disease process and in identifying future specific targets in the development of new treatments for glaucoma.

The identification of risk factors is of the utmost importance in the diagnosis and management of any disease. Unlike genetics, race, and other non-modifiable factors, the use of certain medications that may cause or worsen the pathology can be considered and modified accordingly based on a case-to-case situation of the patient. Wijnants et al. reported an interesting literature review based on the effects of glucocorticoids on IOP [21]. It is well known that about one third of patients are responders, showing elevated IOP after the use of corticosteroids. This literature review showed that most studies reported no significant effects on IOP with the use of intranasal and inhaled glucocorticoids (unless high doses are used). Four out of five studies, however, found elevated IOP levels caused by systemic glucocorticoids, with a possible dose-response relationship. The findings of the current literature regarding use of corticosteroids in patients with either ocular hypertension or glaucoma or patients with risk factors must be kept in mind when managing these patients. If possible, therapy with corticosteroids, especially administered systemically, should be either avoided or limited for brief intervals of time. Patients that do not have alternatives and must continue systemic glucocorticoids for other pathologies need more stringent follow-ups to prevent or promptly treat corticosteroid-induced IOP elevations.

The identification of markers helping in the early glaucoma diagnosis and detection of subtle signs of disease progression is a fascinating field of investigation. In the review article, Murtagh and O’Brien [22] summarize the current knowledge about corneal hysteresis (CH). CH is a relatively new ocular parameter provided by two devices available on the market, which include the Ocular Response Analyzer tonometer (ORA) and the Corneal Visualization Scheimpflug Technology tonometer (Corvis ST). The CH parameter can be defined as the capacity of shock absorption of the cornea, which can be considered as a marker for the ocular compliance. Previous studies have demonstrated that low CH values are a risk factor for the development of glaucoma and marker of its progression, indicating that the CH parameter could play an important role in glaucoma diagnosis and treatment. In the conclusions, the authors suggest that the CH values should be included in an algorithm incorporating IOP, central corneal thickness, and visual field test results, in order to establish the different risk rate for glaucoma development and progression.

The use of intravitreal injections of anti-vascular endothelial growth factor (anti-VEGF) agents to treat various retinal diseases, such as choroidal neovascularization in age-related macular degeneration or high myopia, or macular edema in diabetic retinopathy or retinal vein occlusion, has dramatically increased in the last years. The evaluation of efficacy and safety of the different anti-VEGF agents has been addressed by several authors. Hannape et al. [23] report the clinical results on mid-term impact of anti-VGEF agents on IOP. The Authors retrospectively evaluated the data of 750 patients who were unilaterally injected with anti-VEGF agents; the fellow untreated eye was used as control. An overall slightly significantly increase in IOP between treated and untreated eyes was noticed at 6 months. The comparison amongst different anti-VEGF agents showed that Ranibizumab was associated with a higher rate of clinically significant IOP increase (≥6 mmHg from baseline) at 6 months.

Studies on animal models are of great importance in understanding the impact of ocular hypertension on the ocular structure and function. Mendez-Martinez et al. [24] investigate the influence of chronic ocular hypertension (OHT) on emmetropia in rats. The authors analyzed the effect of an induced mild-moderate chronic OHT on refraction and neuroretina in 260 eyes of young-adult rats over 24 weeks by using optical coherence tomography and electroretinography. The study results clearly show that the OHT accelerates emmetropia in rat eyes towards slowly progressive myopia; OHT also seems to induce an initial increase in structure and function of the neuroretina, which reversed over time.

Glaucoma is more prevalent in adults; however, IOP elevation can also be found in younger age groups. The study entitled “Management of childhood glaucoma following cataract surgery” is a review that evaluates the different treatment options and clinical management strategies reported in current literature for children with glaucoma following cataract surgery [25]. The various therapeutic approaches include medical therapy, angle surgery, glaucoma drainage device implantation, trabeculectomy, and cyclodestructive procedures. A useful flowchart has been provided to guide clinicians in the management of children with glaucoma after cataract surgery.

Two other studies report results obtained with different surgical approaches. Brusini et al. [26] present the results obtained with canaloplasty in a rather large cohort of patients affected with pseudoexfoliation glaucoma with a follow-up period of up to 14 years. Even if this surgical procedure appears to be effective on average, an acute IOP rise was observed in more than 60% of eyes after a long period of satisfactory control. For this reason, the authors conclude that canaloplasty should be either avoided or performed very cautiously in these kinds of patients.

Minimally invasive glaucoma surgery (MIGS) is gaining an increasingly important place in the surgical armamentarium for the treatment of glaucoma. Amongst various MIGS techniques, ab-interno procedures that aims to enlarge Schlemm’s canal facilitating the outflow of aqueous humor through the physiological pathways, are of particular interest. Toneatto et al. [27] show the results of OMNI surgical system alone or in combination with phacoemulsification in 73 patients with open-angle glaucoma. According to this study, this procedure seems to be safe and relatively effective, with a rate of success ranging between 40 and 67.9%.

Another very intriguing topic concerns the possibility of reverseing the structural damage in glaucoma. It is really possible? Park et al. [28] present an interesting study regarding neuroretinal rim recovery after a successful trabeculectomy. This improvement was associated with young age and the amount of IOP reduction obtained, demonstrating that at least a part of neural tissue can undergo a regression of structural damage in the presence of adequate control of IOP.

As guest editors for this Special Issue, we hope you find the manuscripts prepared by our esteemed international colleagues innovative, practical, interesting, and of clinical value.
